# Genomic profiling of collecting duct renal carcinoma

**DOI:** 10.18632/aging.101109

**Published:** 2016-10-26

**Authors:** Sreenivasulu Chintala, Roberto Pili

**Affiliations:** Genitourinary Program, Indiana University-Simon Cancer Center, Indianapolis, IN 46202, USA

**Keywords:** collecting duct renal carcinoma, CDKN2A (p16), SLC7A11 (xCT)

Collecting duct renal carcinoma (CDC) or Bellini cancer is a rare subtype of renal cell carcinoma (RCC), initiated from distal part of the collecting ducts. At the time of diagnosis, about 50% of the patients are found to have metastases and a poor outcome. The response to chemotherapy in CDC patients is modest and overall survival is less than 1 year. Several regimens that include methotrexate, vinblastine, doxorubicin, cisplatin, gemcitabine or paclitaxel have been used to treat CDC patients [1]. Clinical trials with targeted therapies also did not result in a favorable outcome except for selected cases. These findings suggest that there is lack of knowledge on the biology and molecular architecture of these tumors. To understand the mechanisms responsible for drug resistance and to develop novel therapies for this lethal disease we have recently performed whole exome (WES) and transcriptome sequencing (RNAseq) on 7 CDC samples, and we have confirmed specific gene alterations by FISH and immunohistochemistry analysis in a larger cohort (n=16) of cases [2]. We have found frequent loss of *CDKN2A* (62.5%, 10 out of 16) and overexpression of several SLC family transporters including xCT, Cystine transporter (*SLC7A11* gene; 80% 12 out of 15), PROT, Proline transporter (*SLC6A7* gene; 100% 5 out of 5) and GLAST, glutamate and aspartate transporter (*SLC1A3* gene; 80% 4 out of 5) in CDC cases [2]. This is the first report to show overexpression of xCT, a cisplatin resistance associated gene [3], in CDC and to shed some light on the molecular mechanisms responsible for drug resistance. PROT and GLAST involved in transport of Proline and Glutamate and Aspartate have also been showed to be markers associated with drug resistance [4,5]. Our findings suggest that targeting these pathways in CDC may be beneficial and achievable. There are FDA approved drugs that target xCT and PROT, respectively, and could be “repurposed” while waiting to develop more selected agents. For example, sulfasalazine is an anti-inflammatory drug used for colitis and arthritis and targets xCT [3], while benzatropine is an anticholinergic agent used to reduce side effects of antipsychotic treatments and targets PROT [6].

**Figure 1:**
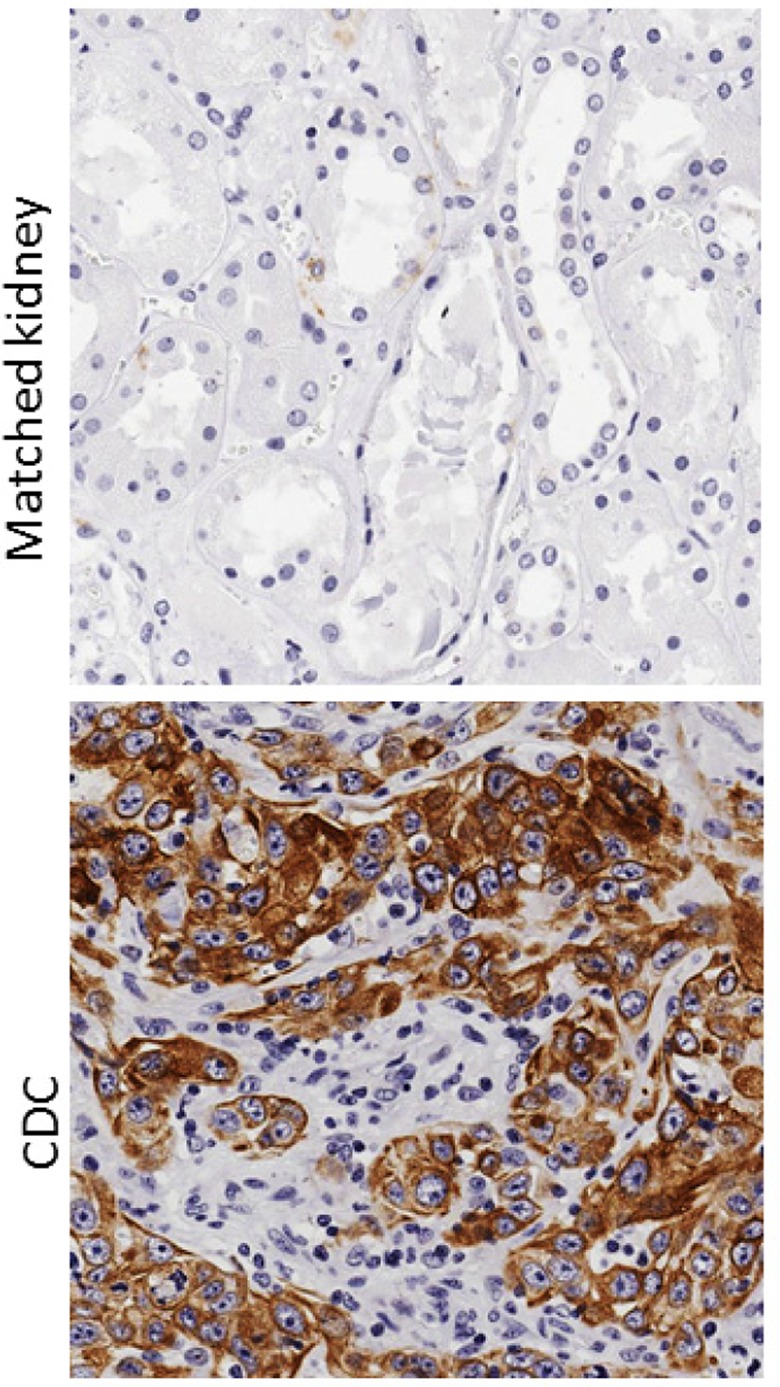
p-mTOR is overexpressed in collecting duct carcinoma tumors Immunohistochemical detection of p-mTOR in FEPE sections. Representative photomicrographs of matched normal kidney (upper panel) and CDC tumor (lower panel).

In order to better understand the biology of CDC, further genomic and proteomic studies are needed. Recently, two other groups have published the genomic profiling of CDC tumors. i) A comprehensive genomic profiling study in 17 CDC patients reporting a common alteration in NF2 (29% 5 out of 17) and suggesting a potential therapeutic role for mTOR inhibitors in CDC [7]. ii) A unique transcriptome sequencing analysis performed on CDC (n=17) comparing with Upper Track Urothelial Carcinomas (UTUC) and other kidney carcinomas [8]. The results from the latter study are quite interesting. The authors discovered that the origin of the CDC is from distal convoluted tubules and possess distinct transcriptome signature among of kidney cancer subtypes. Further, they also showed a metabolic shift in CDC with impaired tricarboxylic acid cycle, pyruvate metabolism and oxidoreductases activity along with the immunogenic response and increased infiltrating lymphocytes especially in the metastatic cases. The authors concluded that CDC exhibits a ‘pathognomonic transcriptomic’ signature with the alteration of immunogenic and metabolic pathways indicating that targeting these pathways might be a therapeutic option for CDC patients. Our findings of *CDKN2A* deletion and overexpression of several oncogenic genes and drug resistance SLC family transporters also suggest that metabolic reprograming is a critical alteration in CDC and targeting these pathways might lead to improve the clinical outcome of patients. Our unpublished data (Figure 1) revealed the overexpression of p-mTOR, a nutritional sensing pathway in 50% (3 out of 6) of CDC cases compared to match normal kidney, again supporting the hypothesis that CDC is a metabolic disease.

In conclusions due to the rarity of the CDC patients, clinicians and researcher working on this disease should get together to pool the available resources and further investigate the molecular alterations by using the currently available advanced Omics technologies. A consensus agreement on the potential treatment options will be beneficial for all the patients affected by this rare but dreadful disease.
